# Phospholipid scramblase 1 as a critical node at the crossroad between autophagy and apoptosis in mantle cell lymphoma

**DOI:** 10.18632/oncotarget.9630

**Published:** 2016-05-26

**Authors:** Katy Mastorci, Barbara Montico, Damiana A. Faè, Luca Sigalotti, Maurilio Ponzoni, Giorgio Inghirami, Riccardo Dolcetti, Jessica Dal Col

**Affiliations:** ^1^ Cancer Bio-Immunotherapy Unit, Department of Translational Research, Centro di Riferimento Oncologico, IRCCS-National Cancer Institute, Aviano (PN), Italy; ^2^ Vita-Salute University San Raffaele, Pathology Unit and Unit of Lymphoid Malignancies, San Raffaele Scientific Institute, Milan, Italy; ^3^ Department of Pathology and CeRMS, University of Torino, Torino, Italy; ^4^ The University of Queensland Diamantina Institute, Translational Research Institute, Brisbane, Australia

**Keywords:** mantle cell lymphoma, phospholipid scramblase 1, autophagy, apoptosis, interferon-α

## Abstract

Mantle cell lymphoma (MCL) is an aggressive haematological malignancy in which the response to therapy can be limited by aberrantly activated molecular and cellular pathways, among which autophagy was recently listed. Our study shows that the 9-*cis*-retinoic acid (RA)/Interferon(IFN)-α combination induces protective autophagy in MCL cell lines and primary cultures reducing the extent of drug-induced apoptosis. The treatment significantly up-regulates phospholipid scramblase 1 (PLSCR1), a protein which bi-directionally flips lipids across membranes. In particular, RA/IFN-α combination concomitantly increases PLSCR1 transcription and controls PLSCR1 protein levels via lysosomal degradation. Herein we describe a new function for PLSCR1 as negative regulator of autophagy. Indeed, PLSCR1 overexpression reduced MCL cell susceptibility to autophagy induced by RA/IFN-α, serum deprivation or mTOR pharmacological inhibition. Moreover, PLSCR1 can bind the ATG12/ATG5 complex preventing ATG16L1 recruitment and its full activation, as indicated by co-immunoprecipitation experiments. The combination of doxorubicin or bortezomib with RA/IFN-α strengthened PLSCR1 up-regulation and enhanced apoptosis, as a likely consequence of the blockade of RA/IFN-α-induced autophagy. Immunohistochemical analysis of 32 MCL biopsies revealed heterogeneous expression of PLSCR1 and suggests its possible implication in the response to anticancer therapies, especially to drugs promoting protective autophagy.

## INTRODUCTION

Mantle cell lymphoma (MCL) is a distinct subtype of malignant B-cell non Hodgkin lymphoma characterized by the t(11;14)(q13;q32) chromosomal translocation, resulting in constitutional overexpression of cyclin D1 and subsequent deregulation of the cyclin D/Rb protein pathway [1, 2]. Despite the critical pathogenic role of this genetic alteration, the t(11;14)(q13;q32) translocation is not sufficient *per se* for the full transformation and malignant evolution of B cells [3, 4]. The complex pathogenesis of MCL integrates alterations in cell cycle regulation, DNA damage response mechanisms, and activation of cell survival pathways [5–9]. Most MCL cases show an aggressive clinical course, with continuous relapse pattern and, often, refractory disease [10]; only, a subset of up to 15% long-term survivors has been identified with a rather indolent clinical course [11, 12]. Despite improved survival data in the younger population, therapy for the elderly or refractory/relapsed patients remains unsatisfactory, and the prognosis quite poor [13, 14].

In the last years, the increased understanding of MCL cell biology led to the use of different therapeutic agents active against this lymphoma, including the proteasome inhibitor bortezomib (BTZ) [15–17], or mammalian target of rapamycin (mTOR) antagonists such as everolimus and temsirolimus [18, 19]. Nevertheless, the response of MCL to these drugs is highly heterogeneous and recent studies showed that the lack of treatment efficacy correlated with induction of autophagy [20, 21]. Several lines of evidence indicate that autophagy can influence the responsiveness to anticancer therapies, since it often functions as a protective mechanism for cell survival under metabolic or drug-dependent stress conditions. In particular, autophagy is correlated to apoptosis and activation of the autophagic machinery can allow the cells to resist and/or elude apoptotic death. Therefore, counteracting autophagy could represent a successful strategy to improve the efficacy of pro-apoptotic chemotherapy [22, 23]. More importantly, the identification of critical regulators of the delicate balance between autophagy and apoptosis could help the design of optimal combination therapy.

Aiming at this goal, we have exploited the features of MCL cell apoptosis induced by the combination of 9-*cis*-retinoic acid (RA) and Interferon(IFN)-α [24], two drugs that may stimulate both pro-apoptotic and autophagic effects in different cellular backgrounds, including lymphocytes [25, 26]. In particular, gene expression profiling approach identified phospholipid scramblase 1 (PLSCR1) as one of the pro-apoptotic genes significantly up-regulated by RA/IFN-α treatment in different MCL cell lines. PLSCR1 is an IFN-inducible protein [27, 28] able to promote rapid transbilayer movement of membrane phospholipids, particularly the exposure of phosphatidylserine on cell surface. Recent data indicate that PLSCR1 may induce apoptotic effects in different cellular systems [29], although no information is currently available on the possible involvement of PLSCR1 in regulating apoptotic and/or autophagic responses in MCL cells. Intriguingly, Huett A. et al. identified PLSCR1 as one of the binding partners of the autophagy-related protein (ATG)12 [30], an ubiquitin-like protein involved in the elongation step of autophagosome formation. The ATG5/ATG12 complex is one of the two ubiquitin-like conjugation systems essential for membrane structure expansion of the phagophore and its full activation is reached through the binding with ATG16L1. Therefore, we investigated the potential interaction between PLSCR1 and the ATG12/ATG5 complex and its possible consequences for drug-induced MCL autophagy. More importantly, analysis of a pilot series of MCL samples disclosed that PLSCR1 is heterogeneously expressed by these lymphomas, suggesting a possible influential relevance of this protein especially as putative predictive marker of clinical response to autophagy-inducer therapeutic agents. These findings deserve to be tested in prospective studies.

## RESULTS

### RA/IFN-α combination promotes protective autophagy in MCL

Previously, we demonstrated that RA/IFN-α treatment induces apoptosis in MCL cells through the inhibition of the PI3-K/Akt pathway [24]. Given the significance of this signalling in the cross-talk between apoptosis and autophagy [31, 32], we investigated the possible relationship between RA/IFN-α-induced cell death and autophagy in MCL cells.

**Figure 1:**
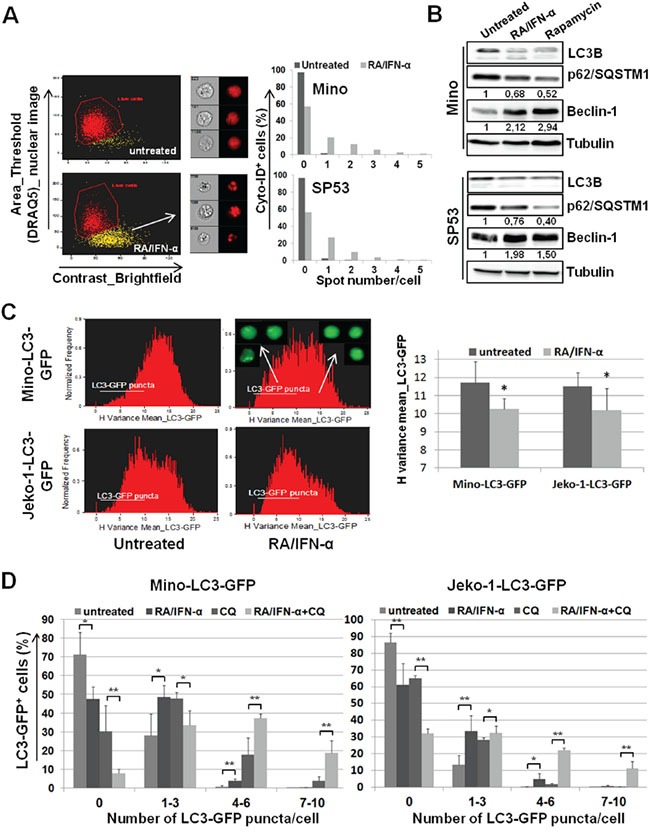
Protective autophagy reduces MCL responsiveness to RA/IFN-α-induced apoptosis **A.** After 72 hours of RA (1 μM)/IFN-α (1000 U/ml) treatment, MCL cells were labelled with the vital nuclear dye DRAQ5, in order to exclude apoptotic cells, and with Cyto-ID autophagy Green dye. 50×10^3^ cells were analyzed for the presence of autophagosomes (green spots). The results are representative of 1 of 2 experiments. **B.** Mino and SP53 cells were treated or not for 48 hours with RA/IFN-α or 1 μM Rapamycin and molecular markers of autophagy were analyzed by immunoblotting. Densitometric analysis of p62 and beclin-1 was reported. **C.** RA/IFN-α treatment (72 hours) decreased H Variance Mean of GFP fluorescence indicating a more clustered LC3-GFP in these samples compared to the untreated ones. The histograms and cell images from IDEAS software on the left are representative of 1 of 3 experiments. On the right, Bars, mean of 3 independent experiments; error bars, SD. *p < 0.05 (T student test) **D.** LC3-GFP puncta formation was evaluated in MCL cell lines after 72 hours of culture in the presence or not of RA/IFN-α. After 48 hours 50 μM CQ was added, where indicated, to block the autophagic flux. Bars, mean of 3 independent experiments; error bars, SD. *p < 0.05; **p < 0,01 (T student test).

**Figure 2:**
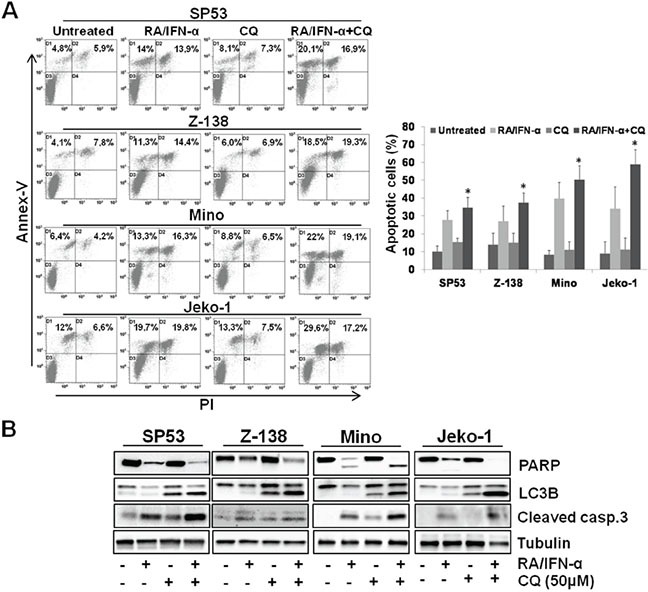
Blockade of RA/IFN-α-induced autophagy enhanced the extent of treatment-induced apoptosis **A-B.** MCL cell lines were exposed to RA/IFN-α combination. After 48 hours 50 μM CQ was added to the culture medium. Apoptosis extent was evaluated at 72 hours of treatment by Annex-V/PI assay and confirmed through PARP and cleaved caspase 3 detection by immunoblotting. Flow cytometry dot plot and immunoblotting analyses are representative of 1 of 3 experiments. On the right, Bars, mean of 3 independent experiments; error bars, SD. *p < 0.05 (T Student test).

As a first step, autophagy was evaluated by multispectral imaging flow cytometry in Mino and SP53 cell lines exposed to RA/IFN-α for 72 hours and labelled with the autophagy Cyto-ID Green dye. This dye specifically accumulates in the autophagosomes allowing the evaluation of the extent of autophagy as number of green fluorescent spots into each single cell. Considering that autophagy usually precedes apoptotic process, the analysis was conducted specifically in living cells, as shown in Figure 1A, excluding apoptotic cells on the bases of nuclear fragmentation. RA/IFN-α treatment induced autophagosome formation as indicated by the increase in the percentage of cells with one or more spots compared to the untreated population. Moreover, molecular markers of autophagy were investigated in the same MCL cells also treated with the mTOR inhibitor rapamycin used as positive control for autophagy induction. Immunoblotting analysis demonstrated Beclin-1 upregulation, p62 downregulation, and LC3B-I decrease with concomitant detection of the lipidated form LC3B-II, in samples treated with RA/IFN-α or rapamycin, consistent with the activation of the autophagic flux (Figure 1B). Likewise, RA/IFN-α significantly (*p < 0.05) increased GFP-LC3 puncta in Mino and Jeko-1 cells infected with the LC3-GFP retroviral expression vector. In particular, these samples were analyzed by exploiting an algorithm of the IDEAS software called “H Variance Mean” (Figure 1C), a cell texture feature that quantifies GFP-LC3 clustering in the presence of high background. In details, lower values of H Variance Mean correspond to more clustered GFP-LC3. Moreover, the addition of the autophagy inhibitor chloroquine further increased the number of LC3-GFP puncta/cell (*p < 0.05; **p < 0.01) (Figure 1D) in RA/IFN-α treated samples compared to the untreated ones. These results, accordingly with the guidelines for autophagy evaluation [33], confirmed the ability of RA/IFN-α treatment to promote autophagy. Notably, inhibition of RA/IFN-α-dependent autophagy with chloroquine markedly enhanced the extent of apoptosis induced by RA/IFN-α (Figure 2A-2B) indicating that in this setting autophagy plays a protective role.

### RA/IFN-α induces PLSCR1 transcription and controls the stability of this protein *via* lysosomal degradation

**Figure 3:**
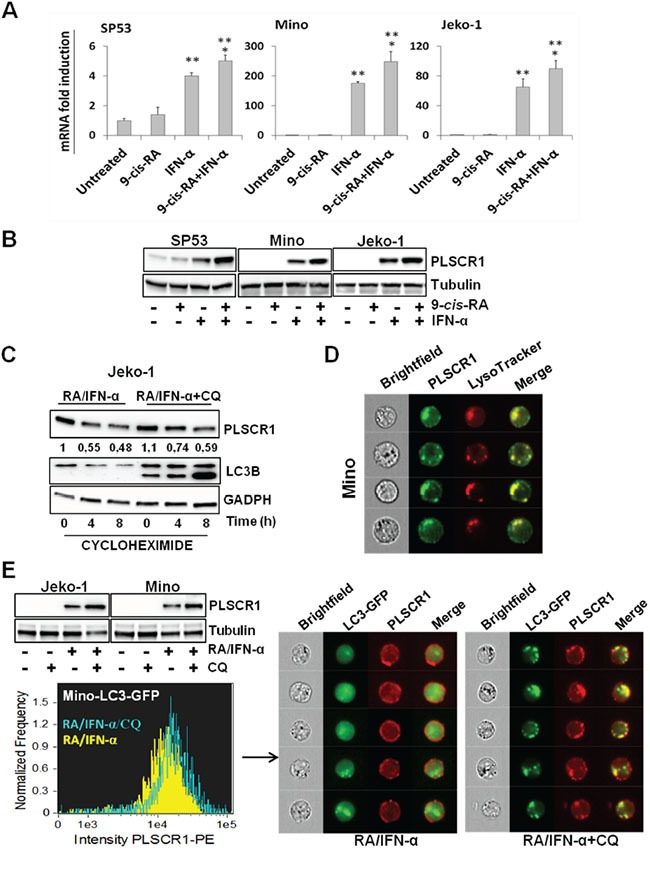
RA/IFN-α combination controls both transcription and protein degradation of PLSCR1 **A-B.** PLSCR1 mRNA and protein were analyzed after 24 hours of treatment with RA (1 μM), IFN-α (1000 U/ml) or their combination. Bars, mean of 3 independent experiments; error bars, SD. **p < 0.01 value relative to untreated and RA-treated samples; *p < 0.05 value relative to IFN-α-treated samples (T student test). The results depicted in B are representative of 1 of 3 independent experiments. **C.** After 48 hour exposure to RA/IFN-α or RA/IFN-α+CQ (50 μM), protein synthesis was blocked by cycloheximide (100 μM) addition in Jeko-1 cell line. Cells were harvested at different time points and PLSCR1 expression was analyzed by immunoblotting. The presence of CQ delayed PLSCR1 protein degradation as demonstrated by densitometric analysis. **D.** RA/IFN-α treatment promoted PLSCR1/Lysotracker co-localization. 10^6^ Mino cells were treated for 2 days with RA/IFN-α and labelled with Lysotracker and anti-PLSCR1 antibody. 50×10^3^ cells were acquired and analyzed with the ImageStreamX instrument. **E.** Blockade of RA/IFN-α-induced autophagy by CQ enhanced PLSCR1 up-regulation in Jeko-1, Mino and Mino LC3-GFP infected cells, and promoted its accumulation into autolysosomes. 10^6^ Mino cells were treated for 2 days with RA/IFN-α and labelled with anti-PLSCR1 antibody. 50×10^3^ cells were acquired and analyzed with the ImageStreamX instrument. PLSCR1/Lysotracker and PLSCR1/LC3-GFP co-localization was measured using the Bright Detailed Similarity Score, a feature of IDEAS software.

Given that autophagy is tightly correlated to apoptosis and profoundly implicated in the responsiveness to anticancer therapies, we reasoned that a more thorough characterization of the mechanisms underlying RA/IFN-α-induced autophagy could be useful to identify markers with a potential predictive value. Gene expression profiling identified PLSCR1 as one of the most significantly up-regulated pro-apoptotic genes in RA/IFN-α-treated MCL cells. These data were validated by real-time qPCR demonstrating the transcriptional induction of PLSCR1 in SP53, Jeko-1 and Mino cells. In particular, treatment with IFN-α alone for 24 hours increased PLSCR1 mRNA levels, and, more interestingly, RA significantly enhanced PLSCR1 induction when added to IFN-α (Figure 3A).

Immunoblotting analysis confirmed a corresponding increase in PLSCR1 protein levels after RA/IFN-α treatment and showed that basal expression of this protein is heterogeneous in the three cell lines studied, with detectable levels only in SP53 cells (Figure 3B). In addition, a prolonged treatment up to 72 hours did not further increase the levels of PLSCR1 expression (not shown), suggesting that RA/IFN-α combination could probably control also protein stability. Therefore, co-treatment with RA/IFN-α and the protein synthesis inhibitor cycloheximide showed that PLSCR1 levels decreased by nearly 50% after 4 hours since cycloheximide addition (Figure 3C). Moreover, the presence of chloroquine together with cycloheximide prevented RA/IFN-α-induced PLSCR1 degradation (Figure 3C) and led to the accumulation of this protein into the lysosomes, as shown by PLSCR1/Lysotracker co-localization (Figure 3D). In keeping with this finding, when chloroquine was used to block RA/IFN-α-induced autophagy, a further up-regulation of PLSCR1 protein levels was observed (Figure 3E). In addition, PLSCR1 transfer into autophagosomes/autolysosomes was detected by multispectral imaging flow cytometry through PLSCR1 co-localization with LC3-GFP puncta (Figure 3E). Taken together, these data indicated that PLSCR1 protein could be degraded by lysosomes and/or autolysosomes during RA/IFN-α-induced protective autophagy and stimulated further investigations to evaluate its potential involvement in the cross-talk between autophagy and apoptosis.

### PLSCR1 prevents autophagy through the binding with the ATG12/ATG5 complex

**Figure 4:**
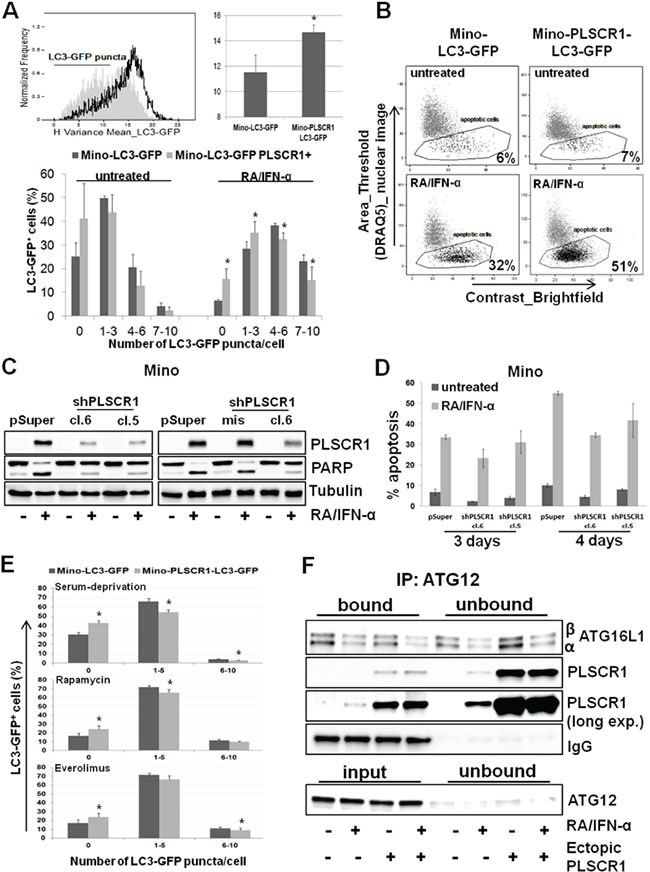
PLSCR1 overexpression reduces cell propensity to undergo autophagy **A.** PLSCR1 overexpression inhibited LC3-GFP clustering and puncta formation after 72 hours of RA/IFN-α treatment; filled curve refers to Mino LC3-GFP, empty curve refers to Mino LC3-GFP overexpressing PLSCR1. In the upper panel autophagy was evaluated as H variance mean. Bars, mean of 3 independent experiments; error bars, SD. In the lower panel, autophagy extent was measured by the counting of LC3-GFP puncta in untreated or treated cells, 50 μM CQ was added to block the autophagic flux after 48 hours of culture. Bars, mean of 5 independent experiments; error bars, SD. *p < 0.05 (T Student test). **B.** Ectopic PLSCR1 concomitantly increases the extent of apoptotic response. The results depicted in B are representative of 1 of 2 independent experiments. **C-D.** PLSCR1 knock down reduced the apoptotic cell fraction after 3 days of exposure to RA/IFN-α combination. Bars in D, mean of 3 independent experiments; error bars, SD. **E.** PLSCR1 overexpression impaired autophagy induction by serum deprivation or pharmacological mTOR inhibition. Cells were starved O.N. or treated with 1 μM Rapamycin or 1 μM Everolimus; after 24 hours 50 μM CQ was added to each sample for 1 day more. 10^6^ cells were analyzed for LC3-GFP puncta counting. Bars, mean of 3 independent experiments; error bars, SD.*p < 0.05 (T Student test). **F.** PLSCR1 protein co-immunoprecipitated with ATG12/ATG5 complex. ATG12 was immunoprecipitated from 150 μg of total lysate and the samples were separated by SDS-PAGE. Expression of the indicated proteins was detected by immunoblotting. Given the overlapping between ATG12 and immunoglobulin heavy chain, ATG12 immunoblotting was performed in the input samples and in the unbound fractions. The absence of the protein in the supernatants indicates indirectly the efficiency of the immunoprecipitation. The results are representative of 1 of 3 independent experiments.

To assess the potential contribution of PLSCR1 to RA/IFN-α-triggered autophagy in MCL cells, we generated a cell line co-expressing ectopic PLSCR1 and LC3-GFP. As shown in Figure 4A, PLSCR1 overexpression significantly (*p < 0.05) decreased the formation of LC3-GFP puncta in RA/IFN-α treated cells (Figure 4A) with a concomitant increase of apoptotic cell fraction (Figure 4B). These results support an inhibitory function of PLSCR1 in the activation of the autophagic cascade and are consistent with a protective role of autophagy in the context of RA/IFN-α-induced apoptosis. To confirm the role of PLSCR1 in mediating MCL cell responses to RA/IFN-α treatment, the protein was knocked down using a short hairpin RNA (shRNA) expression vector containing a specific sequence targeting PLSCR1 mRNA (shPLSCR1) (Figure 4C). On the contrary, Mino cells infected with the empty vector or containing a mismatched sequence (shPLSCR1mis) expressed similar levels of the protein after the exposure to the treatment (Figure 4C). Notably, PLSCR1 silencing decreased the extent of apoptosis induced by RA/IFN-α, as shown by PARP cleavage and the detection of apoptotic cells by Annexin V/7-AAD staining (Figure 4C-4D). Similar results were obtained in SP53 and Jeko-1 cells (Supplementary Figure S1).

Interestingly, PLSCR1 overexpression impaired the ability of MCL cells to undergo autophagy induced also by serum starvation or by treatment with the mTOR inhibitors rapamycin (1 μM) and everolimus (1 μM) (Figure 4E and Supplementary Figure S2), while concomitantly improved cell responsiveness to the anti-proliferative activity of both these drugs (Supplementary Figure S2).

Recently, Huett A. et al., by exploiting a novel hybrid yeast-human network analysis, identified PLSCR1 as one of the binding partners of ATG12 [30], an ubiquitin-like protein involved in the elongation step of autophagosome formation. We therefore evaluated the ability of PLSCR1 to bind ATG12 by co-immunoprecipitation assay. To this end, ATG12 protein was immunoprecipitated from plain and PLSCR1-expressing Mino cell lysates treated or not with RA/IFN-α for 72 hours. Immunoblotting analysis demonstrated that a fraction of PLSCR1 was bound to ATG12/ATG5 conjugates in Mino plain treated cells and this interaction seems to interfere with ATG12/ATG5/ATG16L1 complex formation (Figure 4F and Supplementary Figure S3). Moreover, in Mino cells overexpressing PLSCR1, the bound fraction of ATG16L1, especially the α isoform, was markedly lower than that detected in Mino plain cells (Figure 4F and Supplementary Figure S3). Overall, these results suggest that PLSCR1 may play an inhibitory role in the autophagic process likely interfering with ATG12/ATG5/ATG16L1 complex formation and phagophore elongation.

### Bortezomib and doxorubicin enhanced RA/IFN-α-dependent apoptosis by impairing autophagy induced by this drug combination

**Figure 5:**
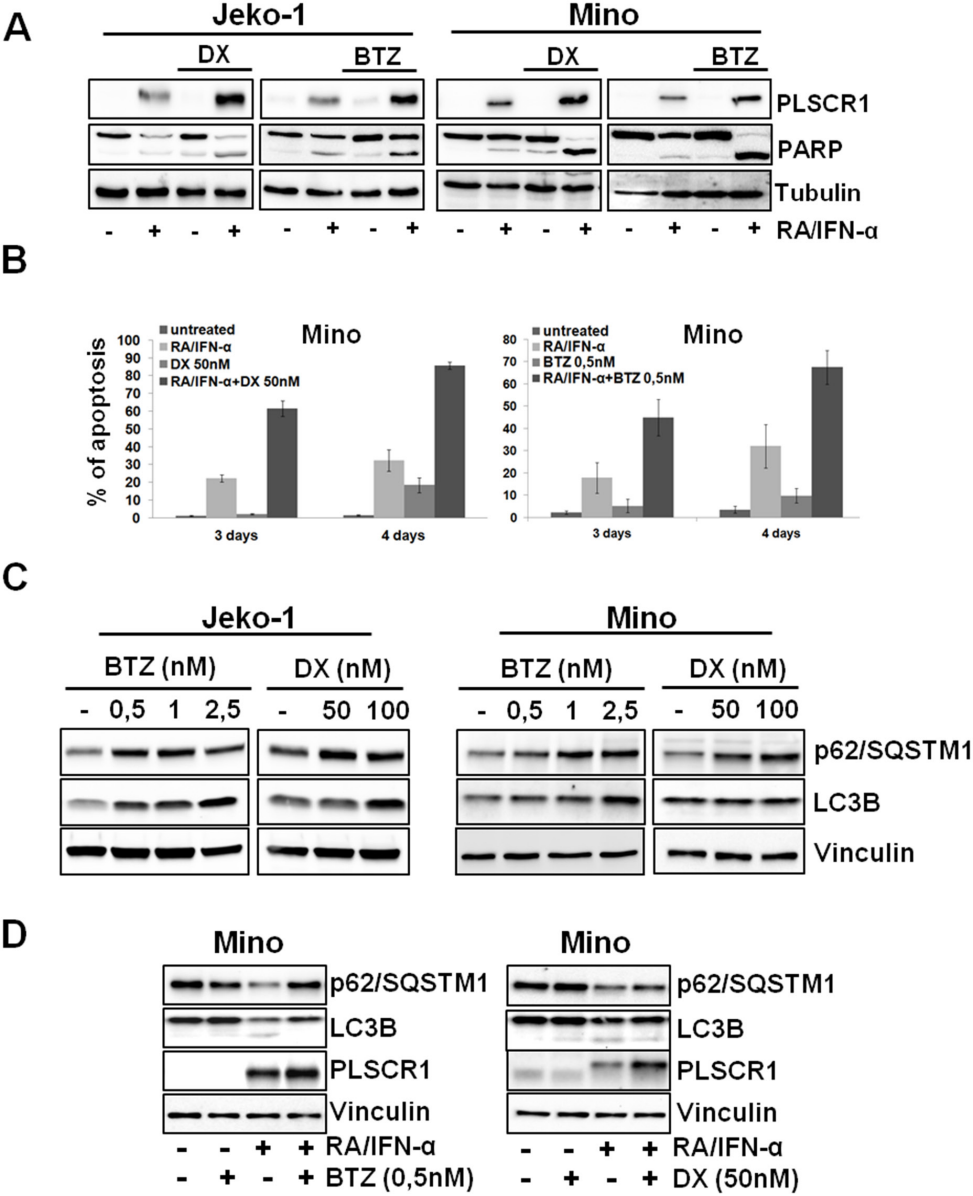
BTZ and DX enhances RA/IFN-α-induced apoptosis through PLSCR1 up-regulation and counteracting treatment-dependent autophagy **A-B.** After 48 hours of RA/IFN-α treatment, DX (50 nM) or BTZ (0.5 nM) were added to the culture medium for additional 24 and 48 hours. PLSCR1 expression and concomitant PARP cleavage were analyzed at fourth day since the beginning of treatment. Apoptosis was analyzed by Annexin-V/PI staining. Bars, mean of 3 independent experiments; error bars, SD. **C.** MCL cell lines were treated for 24 hours with different concentrations of BTZ or DX and the protein levels of the indicated autophagy markers were evaluated by immunoblotting. The results are representative of 1 of 3 independent experiments **D.** Addition of DX or BTZ to 48-hour RA/IFN-α pre-treated Mino cells prevented p62 degradation and LC3B lipidation. Data are representative of 1 of 2 independent experiments.

**Figure 6:**
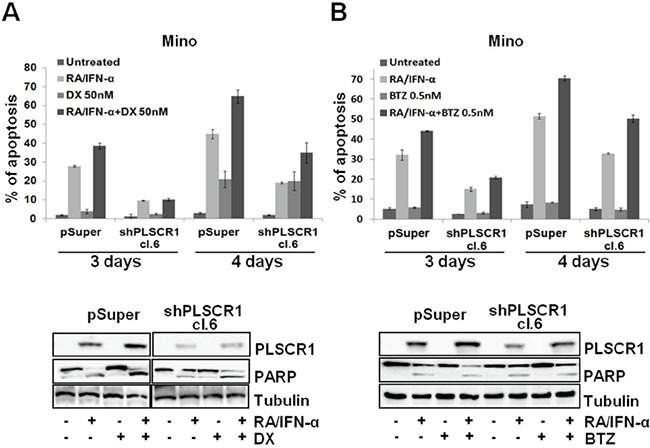
PLSCR1 knock down reduces MCL cell sensitivity to the additional pro-apoptotic effect of DX and BTZ with RA/IFN-α **A-B.** Mino cell lines infected with empty vector or shPLSCR1 were treated for 48 hours with RA/IFN-α, successively DX (50 nM) or BTZ (0.5 nM) were added to the culture medium for additional 24 and 48 hours. PLSCR1 expression and concomitant PARP cleavage were analyzed at fourth day since the beginning of treatment. Apoptosis was analyzed by Annexin-V/7-AAD staining. Bars, mean of 3 independent experiments; error bars, SD.

As shown in Figure 5A, the proteasome inhibitor Bortezomib (BTZ) and Doxorubicin (DX), two drugs currently employed in MCL management, are able to increase RA/IFN-α-induced PLSCR1 expression in MCL cells. In particular, Mino and Jeko-1 cells, in which PLSCR1 was previously induced by 48-hour pre-treatment with RA/IFN-α, were cultured in the absence or presence of sublethal doses of DX (50 nM) or BTZ (0.5 nM) for 24 and 48 hours. The addition of both DX or BTZ enhanced PLSCR1 protein levels (Figure 5A) concomitantly with an increase in apoptosis extent (Figure 5A-5B). To assess whether these results are a consequence of autophagy involvement, we analyzed the effects of BTZ and DX on the autophagic markers p62 and LC3B in Jeko-1 and Mino cells. Immunoblotting analysis revealed that both drugs promoted the accumulation of p62 and LC3B-1 consistently with a blockade of the autophagic flux (Figure 5C). More interestingly, when these two drugs were used in combination with RA/IFN-α, they impaired protective autophagy induced by this treatment with the concomitant PLSCR1 up-regulation coupled with enhanced pro-apoptotic effects (Figure 5D). Furthermore, PLSCR1 knock down decreased the extent of apoptosis in cells pre-treated with RA/IFN-α and successively exposed to DX (Figure 6A) or BTZ (Figure 6B). These findings show that DX and BTZ are able to enhance RA/IFN-α-induced apoptosis by further up-regulating PLSCR1 levels and decreasing RA/IFN-α-promoted autophagy.

### PLSCR1 is heterogeneously expressed in MCL and its expression is inducible by doxorubicin

**Figure 7:**
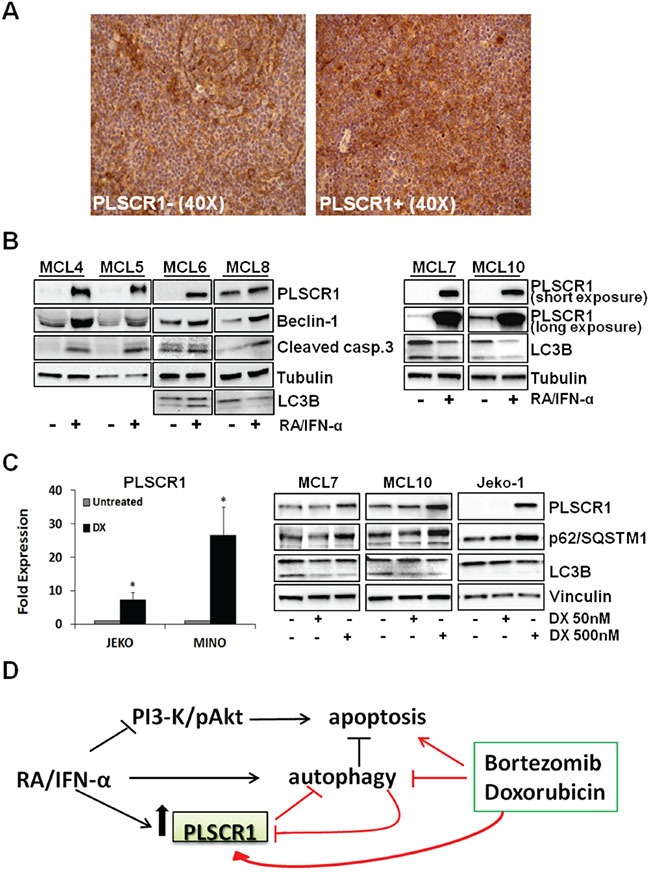
PLSCR1 expression in primary MCLs **A.** Immunohistochemistry analysis showed heterogeneous expression of PLSCR1 in MCLs. Representative negative (on the left) and positive (on the right) samples are reported. **B.** RA/IFN-α treatment (2 days) induced PLSCR1 expression in 6 MCL primary cultures concomitantly with triggering of both autophagy (monitored by beclin-1 accumulation and LC3B) and apoptosis (caspase 3 cleavage). **C.** 24 hour DX exposure (500 nM) up-regulated PLSCR1 mRNA in Mino and Jeko-1 cell lines. Bars, mean of 3 independent experiments; error bars, SD. (*p < 0.05 T Student test). On the right an increase in PLSCR1 protein levels was shown in Jeko-1 cell lines and MCL primary cultures together with a reduction of lipidated LC3B II and p62 accumulation. **D.** Schematic model of RA/IFN-α, DX, BTZ and PLSCR1 interconnection.

Considering the involvement of PLSCR1 in the interconnection between autophagy and apoptosis in MCL and the observed variability of its basal expression in the cell lines included in this study, we analyzed 32 MCL biopsies for the expression of PLSCR1. By immunohistochemical analysis we identified 7 samples (22%) in which PLSCR1 expression was detectable in 10% or more of neoplastic cells and 25 cases (78%) in which it was expressed by less than 10% of tumor cells or was absent. Thus, using the upper quartile method, we calculated 10% as cut-off to subdivide the samples in positive and negative for PLSCR1 expression. The relatively broad range of percentages of PLSCR1 positive cells observed is consistent with an evident intra-tumor and inter-patient heterogeneity (Figure 7A). The analysis of a published microarray dataset (GDS4984) showed different levels also of PLSCR1 mRNA in 38 MCL untreated samples (Supplementary Figure S4).

Expression of the Ki-67 proliferation marker was not significantly different between PLSCR1 positive and negative samples (p > 0.05; two-tailed independent non parametric Mann-Whitney U-Test). Similarly, the expression of cleaved-caspase 3, as a marker of apoptotic cells, was also comparable between PLSCR1 positive and negative MCL cases. These findings are consistent with the observation that PLSCR1 overexpression in Mino cells did not confer any noticeable proliferation advantage (Supplementary Figure S2) nor increased basal apoptosis (Figure 4B), although it resulted in resistance to drug-induced autophagy.

Immunoblotting experiments carried out with 6 MCL primary cultures (MCL4, MCL5, MCL6, MCL7, MCL8, and MCL10) confirmed the heterogeneity of PLSCR1 basal levels in this lymphoma, and showed a marked induction of PLSCR1 expression by RA/IFN-α combination in all samples (Figure 7B). Notably, PLSCR1 up-regulation correlated with a pro-apoptotic effect shown by the increased expression of cleaved caspase-3. Autophagy induction by RA/IFN-α treatment in primary MCL cells was also demonstrated by the accumulation of Beclin-1 and the decrease of LC3B-I (Figure 7B).

Intriguingly, a recent study showed that anthracyclines are able to trigger type I IFN signalling in murine sarcoma, mammary carcinoma and leukemia cell lines, including the transcription of several Interferon stimulated-genes (ISGs) and also of PLSCR2 [34]. These findings prompted us to assess whether anthracyclines were also able to up-regulate PLSCR1 in MCL cells. Although low doses (50 and 100 nM) of DX did not influence PLSCR1 expression in MCL cell lines (not shown), when DX was used at 500 nM a significant increase in PLSCR1 mRNA (*p < 0.05) and protein levels was detectable (Figure 7C). Notably, also the exposure of primary MCL cells from MCL7 and MCL10 to DX at 500 nM, but not at 50 nM, increases PLSCR1 protein levels (Figure 7C). Moreover, immunoblotting analysis of the autophagic markers LC3B and p62 in the same primary MCL cells revealed that, as in Jeko-1, DX promoted the accumulation of p62 and of LC3B-I, in keeping with a block of autophagic flux (Figure 7C). In summary, PLSCR1 is heterogeneously expressed by these lymphomas and chemotherapeutic regimes currently used in MCL management, including anthracyclines, could induce PLSCR1 expression suggesting a potential role of this protein in mediating tumor response to anticancer therapies.

## DISCUSSION

Protective autophagy is known to be a cell survival mechanism that impairs chemotherapy efficacy. Recently, several preclinical studies and clinical trials have evaluated the combination of autophagy inhibitors with conventional anticancer drugs as new therapeutic strategy in different tumors [35–37]. At present, however, only few studies have investigated the possible role of protective autophagy in MCL, but data accumulated so far convincingly demonstrated the critical involvement of this process in the responsiveness to everolimus, flavopiridol, and anti-CD74 monoclonal antibody milatuzumab [20, 38, 39].

In line with these findings, the identification of molecular actors involved in treatment-induced autophagy could be useful to drive the most appropriate therapeutic approach. In the present study, we demonstrate that the pro-apoptotic RA/IFN-α combination induces protective autophagy in MCL cells, and that autophagy inhibition with chloroquine enhances the extent of RA/IFN-α-induced apoptosis. Notably, characterization of the interplay between autophagy and apoptosis in RA/IFN-α-treated MCL cells allowed the identification of PLSCR1 as a novel regulator of the autophagic process. In fact, we provide evidence indicating that this protein is able to counteract RA/IFN-α-, rapamycin-, and everolimus-induced autophagy while simultaneously enhancing MCL cells sensitivity to the antitumor activity of these drugs. Moreover, according to the recent hybrid yeast-human network analysis [30], we showed that PLSCR1 can physically bind the ATG12/ATG5 complex, preventing ATG16L1α recruitment and thereby inhibiting the correct execution of the autophagic process. Interestingly, when the autophagic flux is active in RA/IFN-α-treated MCL cells PLSCR1 protein undergoes lysosomal/autolysosomal degradation.

We demonstrate that PLSCR1 is inducible also in primary MCL cells by RA/IFN-α concomitantly with autophagy and apoptosis promotion. In addition, PLSCR1 expression was detected by immunohistochemistry in lymphoma cells of a fraction of MCL biopsies, although with a broad heterogeneity both inter- and intra-patient. Consistently with *in vitro* findings obtained in MCL cells overexpressing PLSCR1, no correlation was found between PLSCR1 expression and Ki-67 or cleaved caspase-3 in the MCL biopsies investigated, indicating that PLSCR1 *per se* does probably not play a role in regulating MCL cell proliferation or survival. Our results rather suggest that PLSCR1 expression could be correlated to a decreased propensity of tumor cells to undergo autophagy. As a practical implication, these results may provide the rationale to investigate PLSCR1 protein expression in a larger series of MCL patients to assess possible correlations with the response to treatment with drugs, such as everolimus or temsirolimus, whose efficacy is limited by autophagy. Intriguingly, PLSCR1 protein was found overexpressed in colorectal cancer (CRC) and in the corresponding liver metastasis as well [40, 41] and available data suggest that this protein may be involved in tumorigenesis and tumor progression [42], but a possible correlation with autophagy is not currently investigated. Notably, the biological and clinical significance of autophagy in CRC are still poorly understood given that some studies reported contradictory results [43–45]. PLSCR1 expression was also implicated in ovarian cancer response to arsenic trioxide, a pro-apoptotic and pro-autophagic agent [46]. These and our findings stimulate further investigation aimed at elucidating the role of PLSCR1 in autophagy control in both hematologic and solid tumors.

The most common autophagy inhibitors presently used in clinical trials are chloroquine and hydroxychloroquine, antimalarial drugs that abolish the degradative activity of lysosome. Nevertheless, high doses and/or prolonged treatments with chloroquine are burdened with several side effects. Other putative inhibitors, such as bafilomycin A1, pepstatin A and monensin, were studied for their anti-tumor effects *in vitro* and *in vivo*, unfortunately showing an unsatisfactory specificity for the autophagy targets. Accordingly, exploiting the anti-autophagic effects of drugs already-in-use in chemotherapeutic regimens could provide the rationale to optimize current schedules of treatment or develop novel drug combinations. It is worth mentioning in this respect that sublethal doses of both BTZ and DX used in our study can impair RA/IFN-α-dependent protective autophagy, concomitantly enhancing the pro-apoptotic activity of these drugs, effects that are probably correlated with PLSCR1 up-regulation (Figure 7D). In line with our findings, BTZ was recently shown to block cisplatin-induced autophagy and improved the anticancer effects of this drug, although information on the possible involvement of PLSCR1 is lacking [47]. As a further support to the likely broad involvement of PLSCR1 in inhibiting autophagy we also observed that DX, when used as single agent, induces PLSCR1 expression in MCL cells. These result are consistent with the recent demonstration that the pro-apoptotic effects of anthracyclines, and particularly of DX, are correlated to the activation of type I IFNs-dependent signalling in cancer cells [34].

In summary, the present work describes a new function of PLSCR1 as a possible negative regulator of autophagy and suggests a potential involvement of this protein in MCL response to anticancer therapy, especially to autophagy-inducer agents. In addition, our results highlight the importance to re-evaluate presently used chemotherapeutic agents for their capacity to induce or abrogate autophagy, in order to optimize their employment in the design of novel and/or more effective drug combinations in cancer treatment.

## MATERIALS AND METHODS

### Patient samples and cell lines

Thirty-two patients with MCL were diagnosed according to WHO lymphoma classification [48]. The study was performed in accordance with protocols approved by the local ethical committee, and all patients gave their informed consent. Mononuclear cells of 6 patients (Supplementary Table S1) were isolated from unicellular suspension obtained from mechanically minced lymph nodes or spleen. Cells were re-suspended and enriched MCL samples (>70% MCL) were cryopreserved in 10% DMSO until further study. Mino, SP53 and Jeko-1 cell lines, all carrying the t(11;14)(q13;q32) translocation, were generously contributed by Dr. Raymond Lai, University of Alberta and Cross Cancer Institute (Edmonton, Alberta, Canada). Z-138 cell line was generously contributed by Dr Bertoni F., IOSI Oncology Institute of Southern Switzerland, (Bellinzona, Switzerland). Cell lines were authenticated in our lab by fingerprinting (Power Plex 1.2, Promega) in January 2011 and in March 2015. All cells were cultured in RPMI 1640 supplemented with 10% heat-inactivated fetal calf serum (FCS; Lonza), 100 U/ml penicillin, 100 μg/ml streptomycin, and 20 mM L-glutamine (Sigma), and maintained in a humidified 5% CO_2_ incubator at 37°C.

### Antibodies and reagents

Rabbit anti-PLSCR1 antibody was from Genetex; mouse anti-PLSCR1 (1E9), and GADPH from Abcam; PARP (F2), Vinculin and β-tubulin from Santa Cruz Biotechnology, cleaved caspase-3, LC3B, beclin-1, SQSTM1/p62, Atg16L1 (D6D5) and Atg12 (D88H11) from Cell Signaling Technology; mouse monoclonal anti-Atg12 (6E5) from Medical & Biological Laboratories CO.; anti-rabbit conjugated-HRP from Bethyl; anti-mouse conjugated-HRP from Perkinelmer; secondary antibodyes for flow cytometry were from Beckman Coulter. Vital nuclear dye DRAQ5 was from Alexis Biochemicals, Everolimus (RAD001) from Selleck, LysoTraker^®^ Deep Red from Life Technologies; G418, Rapamycin, Chloroquine and 9-*cis*-retinoic acid from Sigma. IntronA was purchased from SP Europe, doxorubicin from EBEWE, and bortezomib from JANSSEN-CILAG.

### Immunohistochemistry

Formalin-fixed, paraffin embedded 4μ-thick sections were stained with anti-scramblase 1 monoclonal antibody (ABCAM, Cambridge, UK, clone 1E9) at a dilution of 1:100, after antigen retrieval using TRIS-EDTA at pH9 for 30 minutes at 97°C. Reaction was developed with polymer and counterstained with haematoxylin. The mount of positive neoplastic cells was evaluated semiquantitatively.

### Autophagy detection

Autophagy was detected in MCL cells infected or not with LC3-GFP retroviral expression vector. 10^6^ cells were labeled using the Cyto-ID^®^ Autophagy Detection Kit (Enzo Life Sciences) according to manufacturer's instructions. Cells undergoing autophagy showed green fluorescent countable punctuate structures. Similarly, in cells infected with LC3-GFP retroviral expression vector, the autophagy extent is measurable through the counting of LC3-GFP puncta per cell. 50×10^3^ cells/sample were acquired with the ImageStreamX instrument (Amnis Corporation, Seattle, WA) using the INSPIRE software and, using a specific feature of the IDEAS analysis software, the number of fluorescent spots per cell was evaluated. The “H variance mean” algorithm of the IDEAS software allowed to evaluate the distribution and the texture of fluorescence into the cells also in the presence of high background.

### Apoptosis detection

Apoptosis was evaluated by fluorescence analysis by Annexin V/7-AAD or Annexin V/PI staining and/or by using ImageStreamX technology with DRAQ5 nuclear dye. This technology allows distinguishing between viable and apoptotic cells based only on the nuclear morphology. Flow cytometric analyses were performed on a FC500 flow cytometer (Beckman Coulter, Milan, Italy).

### RNA extraction, cDNA synthesis and real-time quantitative reverse transcription PCR (qRT-PCR) for PLSCR1 mRNA

Total RNA was extracted from 1 to 3×10^6^ cells by QIAGEN RNeasy Mini Kit. Quantification and integrity of mRNA were determined through the Experion Automated Electrophoresis system (BIO-RAD, Hercules, CA, US). 1 μg of RNA was retro-transcribed into cDNA using the ISCRIPT RT OneTube Supermix according to manufacturer's recommendations (BIO-RAD).

qRT-PCR was performed in a Thermal Cycler CFX96 (BIO-RAD) using SsoFast EvaGreen Supermix according to manufacturer's instructions (BIO-RAD). The PLSCR1 specific primers were designed by Primer3 Input software (version 0.4.0), and were synthesized by SIGMA-Aldrich Co. (St Luois, Missouri, US) (primer forward: 5′-AAATCCAAGCTCCTCCTGGT-3′, primer reverse: 5′-TTTGCCAACCACACACTGTT-3′). Specificity control was performed by BLAST alignment tool. Four different housekeeping genes, beta-actin, GAPDH, beta2-microglobulin (β2M), and 18-S were used. Specific primers for 18-S and β2M were kindly provided by BIO-RAD, while the other primers where designed by the software, as above.

Normalized fold expression was calculated with the formula 2^−ΔΔCt^, through the Bio-Rad CFX Manager software.

### PLSCR1 intracellular localization

PLSCR1 localization into lysosomes or autolysosomes was evaluated by multispectral imaging flow cytometry. 10^6^ cells per sample were fixed with 2% of paraformaldehyde and permeabilized with cold methanol. After a wash with PBS containing 0.5% bovine serum albumin (BSA), cells were incubated with mouse anti-PLSCR1 (1E9) antibody at RT for 30 minutes. After two washes, cells were incubated for 30 minutes in ice with PE- or FITC-anti-Mouse secondary antibody. 50×10^3^ cells were acquired with the ImageStreamX instrument and PLSCR1/Lysotracker or PLSCR1/LC3-GFP co-localization was analyzed with IDEAS software using the Similarity Bright Details Score as previously described [24].

### Extracts preparation, immunoblotting and immunoprecipitation

Whole-cell lysate extracts and immunoprecipitated samples were prepared as previously described [49]. Briefly, in immunoprecipitation experiments, 150 μg of proteins were incubated with 5 μg of anti-Atg12 (6E5) mouse monoclonal antibody and 30 μL of protein A-Sepharose CL4B (Amersham International) overnight, centrifuged, and washed three times with lysis buffer. Proteins were fractionated using SDS-PAGE and transferred onto nitrocellulose membranes. Immunoblotting was performed using Clarity™ Western ECL substrate (BIO-RAD) through Chemidoc instrument (BIO-RAD).

### PLSCR1 and LC3-GFP infection

To generate the pQCXIP-PLSCR1 expression vector, the coding sequence of human PLSCR1 was obtained from TrueClone™ human full-lenght PLSCR1 cDNA by PCR, using a 5′ primer containing the BamHI restriction site and the ATG codon, and a 3′ primer including the stop codon linked to the EcoRI restriction site. After BamHI–EcoRI digestion, the PLSCR1 coding sequence product was cloned directionally in the BamHI–EcoRI-digested pQCXIP retroviral vector. pMXs-LC3-GFP Retroviral Expression Vector was purchased from Cell Biolabs. For infection, in brief, infectious supernatant from pQCXIP, pQCXIP-PLSCR1 and pMXs-LC3-GFP retrovirally transfected Phoenix cells were collected after 48 hours and used for three cycles of infections. Upon infection, Cells were selected with puromycin or sorted using FacsARIA III (Beckton Dickinson) for GFP expression. Finally, PLSCR1 expression was evaluated by flow cytometry (90% of positive cells).

### PLSCR1 silencing

Four different shRNA PLSCR1 constructs were obtained by sub-cloning the double-stranded 64-mer oligonucleotide containing the PLSCR1 target sequences (A: 5′-GGACCTCCAGGATATAGTG-3′; B: 5′-CTCTGGAGAGACCACTAAG-3′; C:5′-AGTCTCCTCAGGAAATCTG-3′) or the mismatched sequence (MIS: 5′-GGACGTCCTGGATTTAGTG-3′) into the pSUPER.retro.neo+GFP vector (pSUPER; OligoEngine). Infection was performed as described above. Immunoblotting analysis of transfected Phoenix cells identified the construct shPLSCR1A as the most efficient in protein silencing, therefore we selected this to perform all subsequent experiments. Upon infection, Mino cells were selected with G418 (1 mg/mL) and the infection efficiency was checked through the detection of GFP expression by flow cytometry (97% positive cells).

## SUPPLEMENTARY TABLE AND FIGURES


