# Supplementary material for: Phospholipid scramblase 1 as a critical node at the crossroad between autophagy and apoptosis in mantle cell lymphoma
Source: Oncotarget. 2016 May 26;7(27):41913–28. doi: 10.18632/oncotarget.9630 (PMC5173105; doi:10.18632/oncotarget.9630)
Supplement: Supplementary file 1 [file oncotarget-07-41913-s001.pdf]

Phospholipid scramblase 1 as a critical node at the crossroad between autophagy and apoptosis in mantle cell lymphoma

SUPPLEMENTARY TABLE AND FIGURES

Supplementary Table S1: MCL cases

Case no.	Sex/age, y	Malignant cell (%)	Type	Cyclin D1	Sample analyzed
MCL4	F/72	95	Classical	+	Lymph node biopsy
MCL5	M/50	86	Classical	+	Lymph node biopsy
MCL6	M/64	95	Classical	+	Lymph node biopsy
MCL7	M/67	96	Classical	n.a.	Spleen
MCL8	M/63	93	Leukemic	+	Peripheral blood
MCL10	F/67	87	Classical	+	Spleen

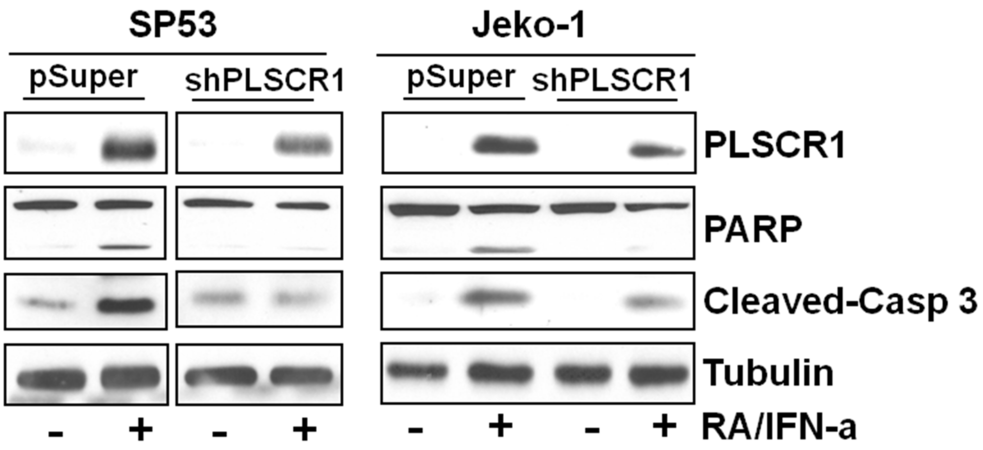

Supplementary Figure S1: PLSCR1 influences RA/IFN-α-induced apoptosis. PLSCR1 knock down reduced the apoptosis extent in Jeko-1 and SP53 cells after 3 days of exposure to RA/IFN-α combination.

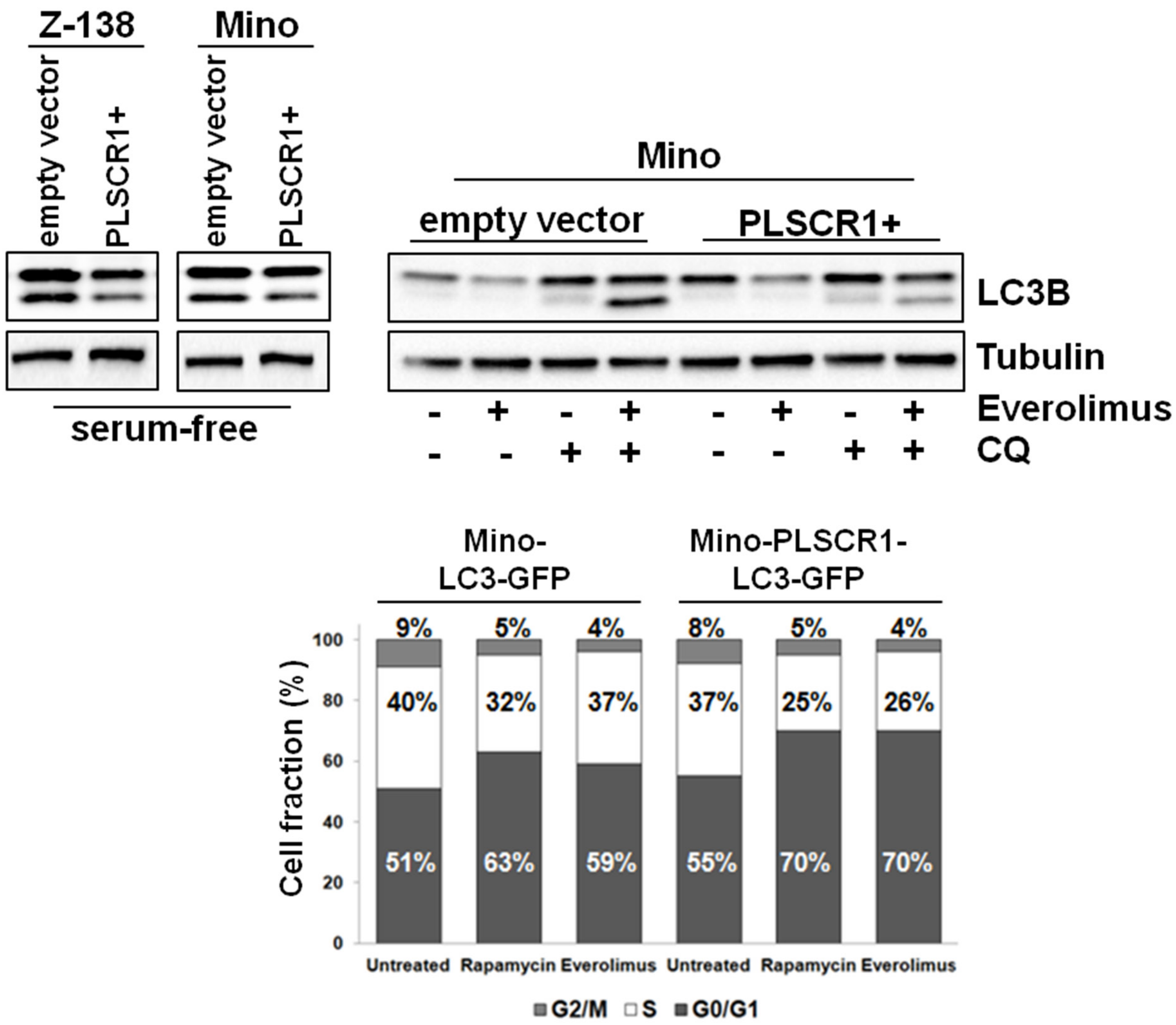

Supplementary Figure S2: PLSCR1 overexpression enhanced MCL cell line response to mTOR inhibition. Mino and Z-138 cells infected with empty vector or ectopic PLSCR1 were starved for 30 min in presence of CQ 50 µM (on the left) or treated with Everolimus 1 µM for 24 h (on the right). The lipidated form LC3B-II was detected by immunoblotting. Mino expressing LC3-GFP or both LC3-GFP and ectopic PLSCR1 were treated for 48 hours with Rapamycin 1 µM or Everolimus 1 µM. Cell cycle was analyzed by FC500 Flow Cytometer after staining with Propidium Iodide in cells permealized with 0,01% NP40. Data are representative of 1 of 2 experiments.

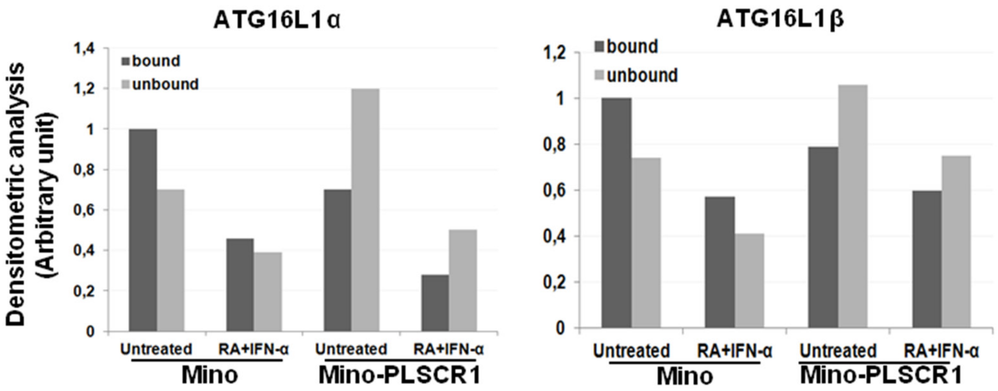

Supplementary Figure S3: PLSCR1-ATG12/ATG5 complex interaction interferes with ATG16L1 recruitment. Densitometric analysis of both isoforms α and β of ATG16L1 protein. The levels of bound and unbound fractions were indicated in arbitrary units and calculated assigning to the first lane of each isoform the value of 1.00.

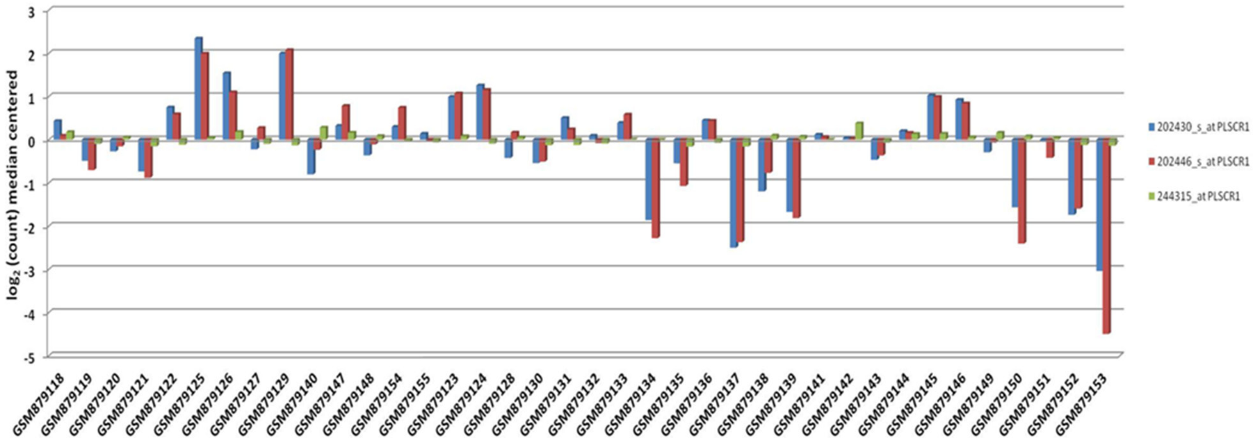

Supplementary Figure S4: PLSCR1 mRNA expression is heterogeneous in MCLs. Expression levels of PLSCR1 in 38 primary untreated MCL patients. For each probe the data were normalized and shown as median-centered Log2 values (GEO accession number GDS4984).
